# 3D Correspondence Between Maxillary Central Incisor Morphology and Facial Shape in Young Adults

**DOI:** 10.3390/dj14010035

**Published:** 2026-01-06

**Authors:** Laura Iosif, Mihaela Pantea, Ana Maria Cristina Țâncu, Teodor-Raul Constantin, Vlad Gabriel Vasilescu, Radu Ilinca, Mirela-Veronica Bucur, Marina Imre, Silviu Pițuru, Lucian Toma Ciocan

**Affiliations:** 1 Department of Prosthodontics, Faculty of Dentistry, “Carol Davila” University of Medicine and Pharmacy, 37 Dionisie Lupu Street, District 2, 020021 Bucharest, Romania; laura.iosif@umfcd.ro (L.I.); mihaela.pantea@umfcd.ro (M.P.); anamaria.tancu@umfcd.ro (A.M.C.Ț.); marina.imre@umfcd.ro (M.I.); 2 Department of Dental Prostheses Technology, Faculty of Dentistry, “Carol Davila” University of Medicine and Pharmacy, 37 Dionisie Lupu Street, District 2, 020021 Bucharest, Romania; teodor-raul.constantin2022@stud.umfcd.ro (T.-R.C.); mirela.bucur@umfcd.ro (M.-V.B.); lucian.ciocan@umfcd.ro (L.T.C.); 3 Discipline of Medical Informatics and Biostatistics, Faculty of Dentistry, “Carol Davila University “ of Medicine and Pharmacy, 4-6 Eforie St, 50037 Bucharest, Romania; 4 Department of Organization, Professional Legislation and Management of the Dental Office, Faculty of Dentistry, “Carol Davila” University of Medicine and Pharmacy, 17-23 Plevnei Str., 020021 Bucharest, Romania; silviu.pituru@umfcd.ro

**Keywords:** maxillary central incisor, facial morphology, three-dimensional imaging, dental esthetics, intraoral scanners, facial scanning, morphometrics

## Abstract

**Backround:** This cross-sectional study aimed to quantify the proportion of 3D tooth–face superimposition in young adults and examine age- and gender-related differences. **Methods:** In 98 dental students, intraoral MCI and facial scans were acquired under standardized protocols, processed in Mesh Mixer v. 3.5.474and Blender v. 4.3.2., and aligned to reference planes for superimposition. Residual tooth volume, reflecting tooth–face correspondence, was computed via Boolean subtraction. Statistical analyses were performed in IBM SPSS Statistics 25 at α = 0.05. **Results:** Total tooth volume (1,626,120.79 ± 210,659.56 × 10^3^) exceeded the superimposed volume by 285,052.34 × 10^3^ (17.53%; 95% CI: 15.84–19.22%; *p* < 0.001), giving a superimposition proportion of 82.6%. Positive correlations between total and remaining tooth volumes were observed overall (ρ = 0.448; *p* < 0.001), in females (ρ = 0.515; *p* < 0.001), and in participants < 21 years (ρ = 0.662; *p* < 0.001). Men had higher total tooth volume than women (1,706,232 ± 151,086 vs. 1,583,561 ± 225,978; *p* = 0.005). **Conclusions:** Volumetric analysis revealed high but incomplete dentofacial correspondence. Larger tooth volumes were associated with greater incongruence in females and younger participants, confirming volumetric size as a determinant of morphological congruence. Results highlight clinically meaningful superimposition, supporting the MCI as a reliable reference for restorative planning and esthetic reconstructions, and emphasize the value of 3D analysis for precise dentofacial evaluation and individualized esthetic planning.

## 1. Introduction

Drawing on the Glossary of Prosthodontic Terms (2023), dental esthetics is defined as the application of esthetic principles to natural or artificial teeth and restorations [1]. In a scientific and clinical context, dental esthetics represents the integration of objective design principles—such as form, proportion, symmetry, and color—with facial morphology to achieve visual harmony. Rather than constituting a distinct specialty, it represents a core objective across multiple dental disciplines, including restorative dentistry, orthodontics, prosthodontics, periodontics, and oral and maxillofacial surgery [2], with the esthetic smile serving as a unifying outcome.

While teeth have long been recognized for their functional and aesthetic contributions to facial appearance [3], contemporary dental esthetics has evolved into a structured framework that standardizes the assessment of dentofacial attractiveness, in which the smile remains central, governed by principles of proportion, balance, and integration within the facial complex [4,5].

Contemporary dental esthetics relies on interrelated dimensions—including dentofacial symmetry, proportional relationships, integration of teeth within the smile arc, balance between dental and gingival components, and harmony of tooth morphology and color—among which the maxillary central incisor (MCI) assumes a pivotal role as both a visual anchor and a primary reference for anterior esthetic planning [6], with its alignment relative to facial midlines and reference planes such as the interpupillary, commissural, and nasolabial lines precisely measurable using contemporary digital tools, encompassing three-dimensional (3D) facial scanners, high-resolution intraoral optical scanners, and CAD/CAM-assisted smile design systems, collectively enabling accurate assessment of midline deviations, tooth angulations, and spatial relationships within the dentofacial complex [7].

Dental proportions provide quantifiable guidance for achieving anterior esthetic harmony, with classical frameworks such as the golden proportion, recurrent esthetic dental (RED) proportion [8,9], and golden percentage [10] serving as reference standards for width relationships among central incisors, lateral incisors, and canines, and when integrated with intraoral scanning and digital smile design workflows, they allow clinicians to plan restorations and orthodontic interventions that optimize both function and esthetics. Equally relevant, the smile arc, defined as the consonance between the curvature of the maxillary incisal edges and that of the lower lip during smiling [11,12], can be systematically evaluated through digital photography, 3D facial scanning, and CAD/CAM analysis [13], facilitating visualization of dynamic interactions between incisal morphology and lip movement and supporting treatment planning that preserves natural esthetic relationships, in which the MCI functions as the central determinant of form, position, color, and proportional relationships that guide the appearance of adjacent teeth and the overall perception of the smile [14,15,16], with its morphology constrained by both geometric parameters of the dental arch and functional integration within the stomatognathic system [17,18].

Odontometric analyses grounded in these principles enable reproducible correlations between MCI dimensions and craniofacial structures, including associations between MCI width and nasal dimensions [19], MCI length with nasal bone length and cranial base parameters across varying skeletal malocclusions [20], and MCI width with bizygomatic width, supporting applications such as Berry’s biometric index for gender determination [21], while additional facial predictors including interpupillary distance [22], inner canthal distance [23], and inter-alar width [24] further refine the estimation of ideal MCI morphology, in accordance with the overarching concept of facial-dental harmony originally articulated by Williams’ law [25].

Over time, various methodologies have been developed to investigate this intriguing association, beginning with the subjective visual assessment method performed by clinicians [26], followed by the introduction of specialized instruments such as the Trubyte tooth indicator [27], the use of classical photographic and cast analysis [28], the digital photographic analysis [29], the application of cephalometric analysis [30], and culminating in the adoption of advanced 3D imaging and printing technologies [31], including facial scanning techniques such as photogrammetry and smartphone-based approaches. Yet, despite these advances, a clear research gap remains: traditional morphological theories, including the law of harmony, lack consensus, most studies rely on 2D or linear measurements rather than volumetric 3D analyses, and population-specific data, particularly in Romanian cohorts, are virtually absent, underscoring the need for precise 3D investigations that integrate dental and facial morphologies [32,33].

Considering that contemporary digital technologies—through the integration of 3D oral and facial scanning combined with advanced CAD tools—enable highly accurate quantitative assessment of dentofacial dimensions, ensuring that the MCI maintains its fundamental role as a key element in anterior dental esthetics, and acknowledging the persistent lack of consensus regarding the validity of traditional morphological theories, the present investigation was designed to determine whether a measurable correspondence exists between the 3D morphology of the buccal surface of the MCI and the facial morphology of the same individual within a population of young adults from Bucharest, Romania, while also exploring potential gender-related differences.

In this context, and in recognition of the need to advance beyond conventional 2D and linear measures toward comprehensive volumetric assessment, our study employs an innovative three-dimensional superimposition protocol with automated computational processing, reflecting current trends in dental research toward objectivity, reproducibility, and high-throughput analysis. To our knowledge, no prior studies have systematically applied such an automated volumetric workflow integrating multi-surface alignment with repeatable computation in similar dentofacial investigations, thereby emphasizing the novelty and potential impact of our approach within the field.

The null hypothesis stated that there is no 3D correspondence between the buccal surface morphology of the MCI and the face shape, whereas the alternative hypothesis posited that if a substantial degree of volumetric correspondence exists, complete morphological congruence does not occur.

## 2. Materials and Methods

### 2.1. Study Design and Sampling Procedures

A cross-sectional study was conducted on dental students at the Carol Davila University of Medicine and Pharmacy in Bucharest, Romania, to investigate the correspondence between the morphology of the vestibular surface of the maxillary central incisor (MCI) and facial morphology using 3D scanning procedures. Students (n = 124) enrolled in the first year, the 2024–2025 academic year, in the Faculty of Dental Medicine were invited to participate voluntarily and anonymously, with detailed information provided regarding the study’s purpose, duration, and procedures.

A total of 98 students consented and met the eligibility criteria; they were assessed as a single cohort using consecutive assignment, reflecting the unequal sex distribution of the group (64 females and 34 males), with ages ranging from 18 to 27 years old.

Inclusion criteria required complete visibility of the facial anthropometric landmarks—including nasion, glabella, pogonion, exocanthion, endocanthion, alare, and cheilion—a structurally intact MCI, and absence of dento-alveolar incongruences. Exclusion criteria were defined as follows: for facial scans, the presence of scars, prior prosthodontic or orthodontic treatment involving the MCI, overlapping hair obscuring key facial features, or wearing glasses during imaging; for intraoral scans, rotated teeth, incomplete scans, or lesions involving loss of substance were considered ineligible.

### 2.2. Data Collection and Ethical Considerations

Data were collected through intraoral and facial scanning under standardized conditions, and the resulting digital files were prepared for analysis. Data collection took place over seven sessions, each including 14 students to ensure consistent conditions according to the investigation protocol, and all scans were performed by three examiners. To minimize operator-related variability and potential sources of error, all scans were performed using consistent scanning distances and uniform movement patterns throughout the procedure. Recalibration of the scanner has been made after each scan, and all the scans have been made in the same conditions (room illumination and temperature). The study was conducted in accordance with the ethical principles of the Declaration of Helsinki and was approved by the Ethics Commission of Scientific Research of the “Carol Davila” University of Medicine and Pharmacy, Bucharest, Romania (approval no. 30846/28.10.2025). No incentives or financial compensation were offered.

### 2.3. Study Protocol and Instruments

The scanning procedures were performed in the same room under consistent lighting and temperature conditions at the Interdisciplinary Center of Research and Development in Stomatology, Digital Techniques in Dentistry Laboratory, U.M.F. “Carol Davila”, Bucharest. The room was equipped with standardized lighting and climate control systems to maintain optimal conditions throughout the data collection period. No software updates or hardware modifications were made during data acquisition to ensure consistency. Oral scanning of the subjects was conducted using a Medit i700 Wired Intraoral Scanner (MEDIT Corp., Seoul, Republic of Korea) to capture the upper anterior teeth. Facial scanning was performed with RAYFace (RAY Co., New York, NY, USA) to acquire the facial geometry. All acquired oral and facial scans were exported as stereolithography (STL) files and processed using MeshMixer v. 3.5.474 (Autodesk Inc., San Francisco, CA, USA) and Blender v. 4.3.2. (Blender Foundation, Amsterdam, The Netherlands). Data processing included editing of intraoral and facial scans, superimposition, and face-to-tooth matching analysis. To reduce operator-related variability and improve reproducibility, custom Python scripts were incorporated into Blender to automate editing, alignment, and analysis procedures.

Analyses were conducted on subjects in a static facial position, as the correspondence between the 3D morphology of the central incisor crown and facial shape was assessed under these conditions. While lip dynamics and soft tissue behavior during motion are recognized as important for esthetic perception, including smile expression and dental arch exposure, these aspects were not included in the present protocol, allowing the study to focus on reproducible static measurements while preserving clinical relevance.

**Figure 1:**
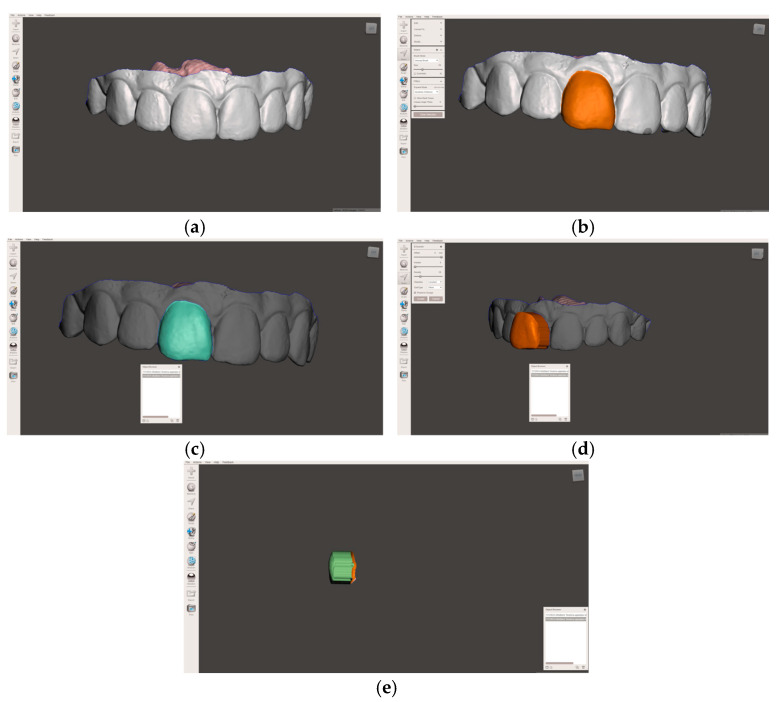
Editing of the intraoral scan: (**a**) importing the intraoral scan of the subject; (**b**) selection of the buccal surface of the MCI; (**c**) optimization and smoothing of the buccal surface selection boundary; (**d**) creating a 3D object by extrusion of the buccal surface by a preset value of 6 mm; (**e**) print screen of extruded object ready for export and alignment in Blender.

The intraoral scan was used to obtain the morphology of the MCI (tooth 1.1, FDI notation). After importing the intraoral scan into Meshmixer, the buccal surface of the tooth was selected using an optimized and smoothed boundary. At this stage, a digital 3D object was generated by extrusion, with the buccal surface of the maxillary central incisor serving as the top surface and an extrusion height adequate for virtual manipulation and accurate superimposition. After several trial runs, the extrusion value was set to 6 mm and recorded as the preset extrusion value for all samples. The virtual 3D object was further exported as an STL file for subsequent import into Blender v. 4.3.2. (Figure 1) software.

**Figure 2:**
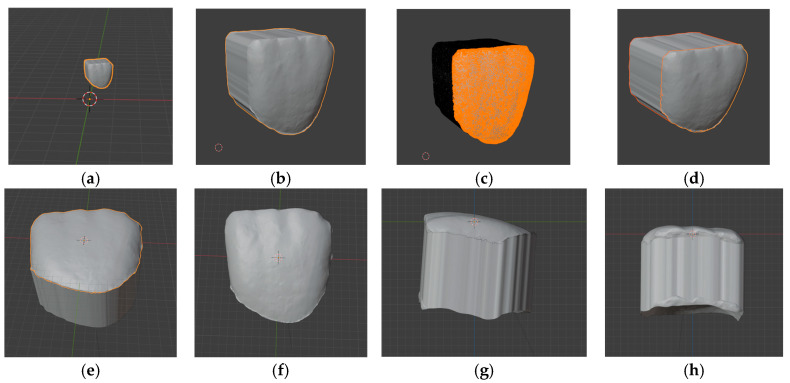
Blender software screen shots of the imported 3D object and alignment of tooth scan in Blender environment: (**a**) importing of the extruded MCI buccal shape into Blender; (**b**) scaling by 15.56 of the extruded object; (**c**) selection of the buccal surface of the tooth from the extruded body; (**d**) separation of the buccal surface and “parenting”; (**e**) setting origin to center of mass; (**f**) alignment with the cursor to center of environment; (**g**) selection of proximal edge midpoint; (**h**) selection of incisal edge midpoint.

Following import into Blender, a reference plane was established for the final superimposition and analysis. The alignment to the reference plane was performed in several steps. First, the imported 3D object had to be magnified (scaled in the 3D software). The elected scaling value was 15.56, corresponding to the mean ratio of bizygomatic width to tooth width [34], and was registered as a preset scaling value for all samples. The buccal surface was then separated and designated as the “parent” for the extruded body to prevent unintended mesh modifications and to preserve the spatial relationship of the buccal surface to the rest of the model (Figure 2).

Next, the origin of the body was set to its center of volume, and the model was centered at the global coordinates (XYZ 0, 0, 0). Three anatomical points were then designated to define the reference plane: the incisal midpoint and the midpoints of the proximal edges. The incisal midpoint was automatically aligned along the Z axis, while the midpoints of the proximal edges were aligned equally within the XY plane. This procedure ensured a standardized orientation of the model for subsequent superimposition and morphometric analysis.

**Figure 3:**
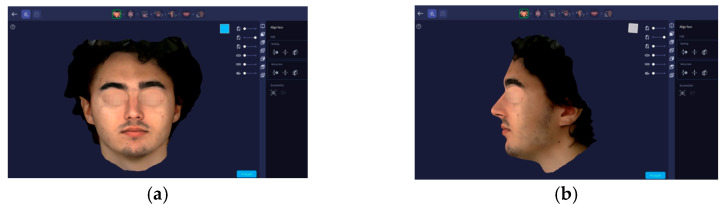
Facial scanning image captions: (**a**) anterior and (**b**) lateral face view seen on RAYFace scanner integrated software.

Facial scanning was performed with a professional RAYFace scanner (RAY Co., New York, NY, USA) to acquire the facial geometry. The subjects were properly positioned on the adjustable chair in order to have the facial geometry recorded. The scanner’s integrated software has the feature to control and standardize the results registered (Figure 3).

**Figure 4:**
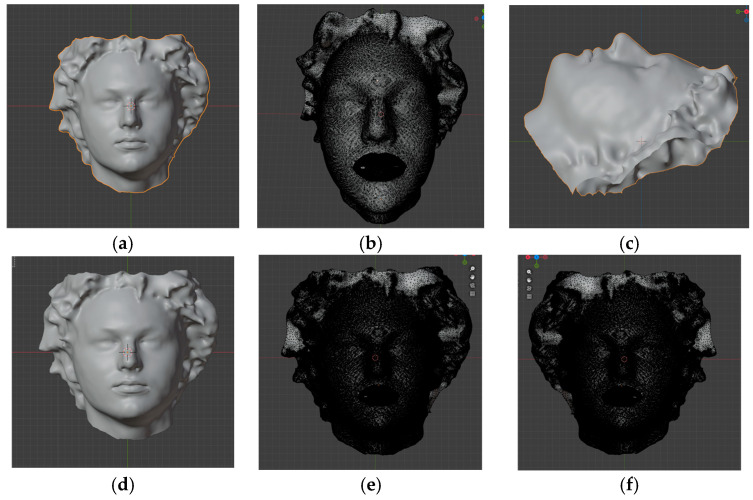
Screen shots in Blender software of importing and alignment of the facial scan to the designated reference plane: (**a**) importing the facial scan into Blender; (**b**) selection of anthropometric landmarks for alignment; (**c**) alignment of glabella and pogonion to the Y axis; (**d**) setting the origin to the center of mass and snapping the model to the environment center; (**e**,**f**) selection of midline vertices for final alignment.

Subsequently, all facial scans were automatically imported and aligned to the selected reference plane. The anthropometric landmarks glabella and pogonion were used to align the scans along the Y axis. The scan origin was set to the center of mass, and the model was centered at global coordinates (XYZ 0, 0, 0). Two midline points (glabella and labial superius) were then used to finalize the alignment, rotating the scan as needed to ensure the midline matched the Y axis (Figure 4).

**Figure 5:**
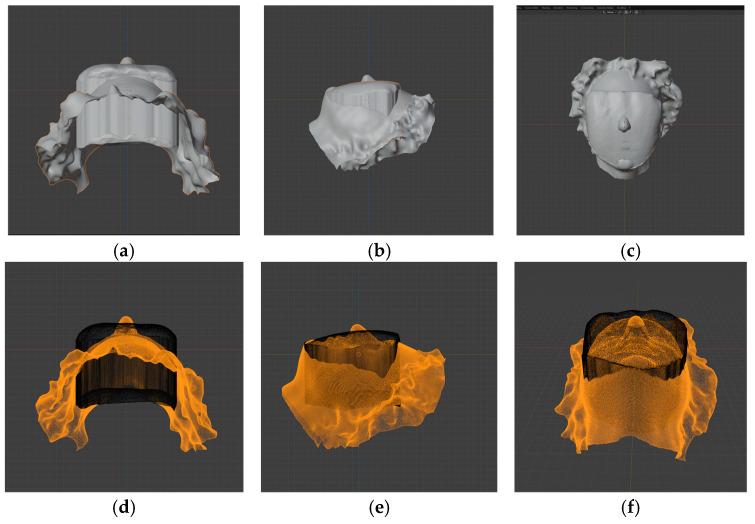
Screenshots in Blender software of the superimposition from different perspectives in solid and mesh renderings of the extruded MCI buccal surface and face: (**a**,**d**) spatial relationship of the incisal edge to the facial profile; (**b**,**e**) spatial relationship of the cervical margin to the pogonion; (**c**,**f**) frontal view of the superimposition.

The resulting Blender scene incorporated the aligned facial scan and the buccal surface of the MCI (Figure 5), along with the extruded object derived from the buccal face. A Boolean operation was used to intersect the extruded object with the facial mesh. From the resulting mesh (face minus tooth), the remaining tooth volume (RTV) was calculated. A smaller remaining tooth volume indicated a closer fit between the tooth and the facial geometry, reflecting that the tooth shape corresponded more accurately to the facial structure.

**Figure 6:**
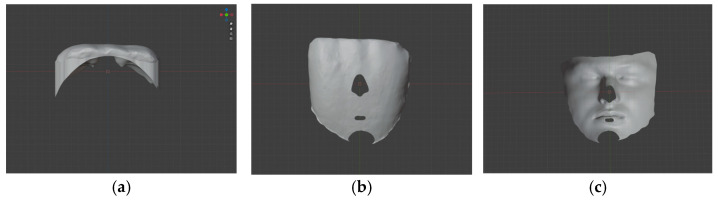
Screenshots in Blender software of the assessment of the remaining tooth volume: (**a**) view along the *Y* axis; (**b**) superior view along the *Z*-axis; (**c**) posterior view along the *Z* axis.

**Figure 7:**
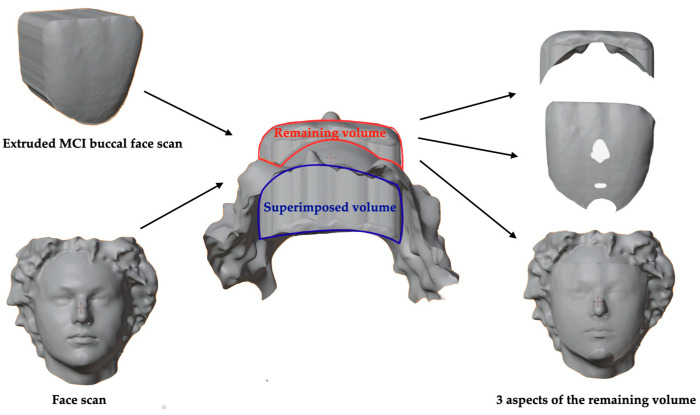
Schematic illustration of superimposed and remaining volume.

For the superimposition procedure, the facial scan was first manipulated so that the Y axis was tangent to both the glabella and menton points, while the tooth was aligned cervico-incisally along the Y axis. For mesio-distal alignment, the X axis was oriented tangent to the zygomatic prominences or the nasal wings, and the most mesial and distal points of the tooth were aligned accordingly. The alignment process was performed sequentially: the facial scan was selected first, followed by the tooth, and a Boolean subtraction was applied to generate the remaining tooth volume (RTV) and calculate the percentage of superimposition (%SI) (Figure 6 and Figure 7).

The mathematical formulas for key outcome variables are as follows:

RTV = V_total − V_superimposed

%SI = (V_superimposed/V_total) × 100

These parameters quantify the degree of three-dimensional correspondence between tooth and face, with lower RTV and higher %SI indicating closer morphological integration.

### 2.4. Data Management and Analysis

All the data from the study were analyzed using IBM SPSS Statistics 25 and illustrated using Microsoft Office Excel/Word 2024. Qualitative variables are expressed as counts or percentages. Quantitative variables are reported as means with standard deviations or medians with interquartile ranges. Normality of the quantitative variables was assessed using the Shapiro–Wilk test. Quantitative variables with normal distribution were compared between age/gender groups using Student’s *t*-test (after verifying the equality of variances using Levene’s test). Quantitative variables with non-parametric distribution were compared between age/gender groups using the Mann–Whitney U test. Quantitative variables with non-parametric distribution were correlated using Spearman’s rho correlation coefficient. Quantitative paired variables with normal distribution were tested between measurements using a paired-sample *t*-test. The threshold considered for the significance level for all tests was considered to be α = 0.05.

## 3. Results

A total of 98 participants were included in the analysis. The sample had a mean age of 20.56 ± 1.29 years (median = 20 years) and comprised 64 females (65.3%) and 34 males (32.7 %). Participants younger than 21 years represented 52.1% of the cohort (Table 1).

The primary endpoint was the within-subject difference between total tooth volume and superimposed tooth volume. A sensitivity/power analysis was performed using the observed precision of the primary estimate (two-sided α = 0.05). For n = 98, the primary paired comparison (mean difference = 285,052.34; 95% CI 257,583–312,521) corresponded to a very large, paired effect size (Cohen’s dz ≈ 2.08), implying an achieved power of >0.999. For the sex comparison in total tooth volume (69 females vs. 29 males), the observed standardized difference was moderate (Cohen’s d ≈ 0.59), corresponding to an achieved power of approximately 0.76. For the reported correlation between total tooth volume and remaining tooth volume (Spearman’s ρ = 0.448; n = 98), the power to detect a correlation of this magnitude was approximately 0.997 (two-sided α = 0.05).

The mean total tooth volume was 1,626,120.79 ± 210,659.56 (median = 1,634,340.50; IQR = 1,452,303.57–1,782,391.84; range = 1,044,349–2,070,316). The mean remaining tooth volume was 285,052.34 ± 137,012.73 (median = 240,659.19; IQR = 193,097.42–336,239.56; range = 95,530–961,702). The mean superimposed tooth volume was 1,341,068.45 ± 195,230.40 (median = 1,355,427.57; IQR = 1,192,257.81–1,471,840.53; range = 811,049–1,717,173) (Table 1, Figure 6). The mean superimposition percentage was 82.64 ± 7.36% (median = 84.70%; IQR = 79.73–87.88%; range = 51.69–93.70%) (Table 1).

**Figure 8:**
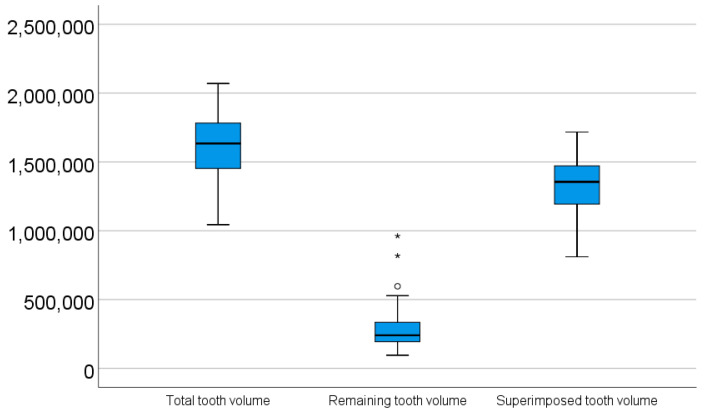
Box-plot figure illustration of all analyzed volumes.

A significant positive correlation of moderate strength was found between total tooth volume and remaining tooth volume (n = 98; Spearman’s ρ = 0.448, *p* < 0.001) (Table 2, Figure 8). The remaining tooth volume showed a non-parametric distribution according to the Shapiro–Wilk test (*p* < 0.001).

A significant difference was found between total tooth volume and superimposed tooth volume (n = 98; *p* < 0.001). Both variables showed a normal distribution according to the Shapiro–Wilk test (*p* > 0.05). The mean difference between the total and superimposed tooth volume was 285,052.34 (95% CI = 257,583–312,521), corresponding to 17.53% (95% CI = 15.84–19.22%) of the mean total tooth volume (Table 3).

However, no significant correlations were identified between age and total tooth volume or superimposition percentage (n = 98; *p* > 0.05). Both age and the volumetric variables showed no significant correlation according to Spearman’s analysis (Table 4). Regarding age groups, no significant differences were found in total tooth volume or superimposition percentage (n = 98; *p* > 0.05). Both parameters showed comparable values across age groups (Table 4).

Regarding gender, total tooth volume was significantly higher in men (1,706,232 ± 151,086) than in women (1,583,561 ± 225,978; n = 98; *p* = 0.005), whereas superimposition percentage did not differ significantly between genders (*p* = 0.526) (Table 5).

A significant positive correlation of high strength was found between total tooth volume and remaining tooth volume in women (n = 69; Spearman’s ρ = 0.515, *p* < 0.001). Remaining tooth volume showed a non-parametric distribution according to the Shapiro–Wilk test (*p* < 0.001) (Table 6). The same results were found in men, with no significant correlation between the total tooth volume and the remaining tooth volume (n = 29; Spearman’s ρ = 0.142, *p* = 0.310). The remaining tooth volume showed a non-parametric distribution according to the Shapiro–Wilk test (*p* = 0.041) (Table 6).

Further, in women, a significant difference was found between total tooth volume and superimposed tooth volume (n = 69; *p* < 0.001). Both variables showed a normal distribution according to the Shapiro–Wilk test (*p* > 0.05). The mean difference between the total and superimposed tooth volume was 285,296.23 (95% CI = 248,004–322,587), corresponding to 18.02% (95% CI = 15.66–20.37%) of the mean total tooth volume (Table 7). In a similar manner, a significant difference was found between the total tooth volume and superimposed tooth volume in men (n = 29; *p* < 0.001). Both variables showed a normal distribution according to the Shapiro–Wilk test (*p* > 0.05). The mean difference between the total and superimposed tooth volume was 284,593.25 (95% CI = 245,379–323,807), corresponding to 16.68% (95% CI = 14.38–18.98%) of the mean total tooth volume (Table 7).

In participants aged < 21 years, a significant positive correlation of high strength was found between total tooth volume and remaining tooth volume (n = 51; Spearman’s ρ = 0.662, *p* < 0.001). Remaining tooth volume showed a non-parametric distribution according to the Shapiro–Wilk test (*p* < 0.001) (Table 8). In participants aged ≥ 21 years, no significant correlation was found between total tooth volume and remaining tooth volume (n = 47; Spearman’s ρ = 0.122, *p* = 0.251). The remaining tooth volume showed a non-parametric distribution according to the Shapiro–Wilk test (*p* < 0.001) (Table 8).

A significant difference was found between the total tooth volume and superimposed tooth volume in the <21 years age group (n = 51; *p* < 0.001). Both variables showed a normal distribution according to the Shapiro–Wilk test (*p* > 0.05). The mean difference between total and superimposed tooth volume was 294,131.51 (95% CI = 253,799–334,463), corresponding to 17.80% (95% CI = 15.36–20.24%) of the mean total tooth volume (Table 9). The total tooth volume was significantly higher than the superimposed tooth volume in the ≥21 years age group (n = 47; *p* < 0.001). Both variables showed a normal distribution according to the Shapiro–Wilk test (*p* > 0.05). The mean difference between total and superimposed tooth volume was 275,011.23 (95% CI = 234,909–315,092), corresponding to 17.28% (95% CI = 14.76–19.80%) of the mean total tooth volume (Table 9).

Overall, after statistical analysis of the 3D superimpositions, the results led us to reject the null hypothesis, which stated that no 3D correspondence exists between the buccal surface morphology of the MCI and facial shape. The results provide supporting evidence for the alternative hypothesis, meaning a substantial degree of volumetric correspondence is observed without achieving a complete morphological congruence.

## 4. Discussion

Dentofacial aesthetics, smile design, and the perception of beauty and harmony have long been central concerns in restorative dentistry and prosthodontics, yet they often remain subjective, depending on the clinician’s experience or the dental technician’s interpretation. With the advent of advanced digital tools, patients are increasingly able to select natural-looking teeth and smiles that match their personal preferences, trying them virtually through 3D digital mock-ups or physical prototypes, while virtual and augmented reality applications can superimpose these designs in dynamic 3D simulations [35,36,37]. These innovations allow for precise 3D analyses of dental and facial structures, improving planning accuracy and patient communication. With the integration of artificial intelligence as a new frontier in digital prosthodontics, facial outcome prediction in completely edentulous patients has gained unprecedented precision and personalization [38,39]. Numerous studies have explored correlations between facial measurements and tooth dimensions to optimize prosthetic design; however, to the best of our knowledge, no comprehensive 3D digital analysis has yet been conducted within the Romanian population. Investigating such population-specific morphological correlations is essential to advance emerging predictive models and to ensure prosthetic rehabilitation that is both functionally precise and aesthetically harmonious with individual facial features.

Our study was designed to evaluate the 3D correspondence between the MCI and overall facial morphology, employing advanced digital technologies to achieve a level of precision unattainable with conventional methods. Although the analysis revealed a notably high degree of superimposition (mean 82.6%), a remaining volumetric discrepancy of approximately 17.5% persisted, thereby demonstrating that complete morphological harmony is seldom achieved in practice. Considering the validation provided by state-of-the-art 3D digital technology, the result strongly supports both working hypotheses: the null hypothesis, which anticipated the absence of perfect superimposition, and the alternative hypothesis, which predicted substantial volumetric correspondence without achieving absolute congruence. Such evidence becomes particularly significant when compared with the findings of Wegstein P.G. et al. [29], who also examined the right MCI using 3D datasets of facial and dental structures but reported only weak, statistically non-significant correlations between face shape and tooth form, with prediction accuracy as low as 18% and Hausdorff distances exceeding 1 mm. In contrast, our protocol avoids reliance on indirect statistical inference by employing volumetric superimposition based on high-resolution 3D scans, offering an objective and reproducible metric of correspondence. This distinction is crucial for clinical application, as volumetric congruence reflects the actual morphological fit relevant to esthetic planning, whereas predictive modeling, even when methodologically advanced, cannot ensure practical accuracy for individualized treatment.

An important observation emerging from our study is the presence of a significant positive correlation between total tooth volume and the remaining volume after superimposition. This finding suggests that MCI’s with larger volume tend to exhibit a less precise morphological fit with the facial contour, which may have implications for individualized esthetic planning. Unlike other recent studies that primarily investigated linear dimensions or tooth shape and reported weak or non-significant correlations with facial parameters [32,40,41,42], our findings introduce a novel perspective by demonstrating a correlation between the total tooth volume of MCIs and the remaining volume after superimposition, which suggest that volumetric size may influence the degree of morphological congruence with facial morphology, an aspect overlooked in earlier 2D or proportion-based approaches. A second noteworthy complementary result is that, beyond this trend, a consistent volumetric discrepancy was present across all cases, indicating that perfect morphological alignment between MCIs and facial morphology is rarely achieved, regardless of tooth size, and emphasizing the inherent complexity of dentofacial integration. This observation is consistent with the prevailing consensus in the literature, as both classical and contemporary studies—starting from Williams’ law of harmony [15] and subsequent works by Wright [43] and Tancu [44] and extending to recent investigations such as those by Dervarič et al. [42] —have repeatedly demonstrated that perfect morphological alignment between maxillary central incisors and facial morphology is rarely achieved, despite methodological refinements and technological advances.

Another dimension explored in our study concerns the potential influence of age on dentofacial relationships, given that all participants were young adults but with a distribution allowing subgroup analysis (<21 years vs. ≥21 years). We considered this perspective clinically relevant because craniofacial growth and dental morphology undergo subtle changes during late adolescence and early adulthood, which could theoretically affect the volumetric congruence between maxillary central incisors and facial morphology with most craniofacial growth being completed by late adolescence—typically around 16–17 years in females and 21–22 years in males [45,46]. Therefore, the age of 21 is commonly regarded as a practical threshold for distinguishing individuals who may still exhibit remaining growth from those considered skeletally mature [47,48]. We examined whether age correlates with key volumetric parameters—total tooth volume, superimposition percentage, and remaining volume—and whether differences appear between the two age groups. No significant correlations were found between chronological age and either the total tooth volume or superimposition percentage, despite the sample covering a broad age range (18–27 years). This observation is consistent with previous evidence indicating that dentofacial dimensions stabilize after pubertal growth, with minimal changes during early adulthood [49,50]. Comparable results were reported by Radia et al. [34] in dental students aged 18–30 years, where no association was found between age and maxillary central incisor proportions.

Initially, stratifying participants into two age groups revealed no significant differences in total tooth volume or superimposition percentages. Subgroup analysis, however, demonstrated a strong positive correlation between the total tooth volume and the remaining volume in participants younger than 21 years, whereas no such association was observed in the older group. This indicates that, in individuals who have not reached full skeletal maturity, larger MCIs tend to exhibit greater volumetric divergence from the facial surface, likely reflecting ongoing adaptive changes in soft tissues or remaining craniofacial remodeling. Conversely, the absence of correlation in the ≥21 group suggests that, once skeletal growth is complete, the relationship between tooth size and facial morphology becomes more stable.

Our results are further supported by recent research, which consistently demonstrates that craniofacial skeletal growth and remodeling persist beyond late adolescence, particularly in males. For example, significant mandibular and midfacial growth continues from ages 16 to 20, with the most pronounced changes between 16 and 18, but measurable growth is still present through 18–20 years [51,52,53]. These studies also show that overall mandibular growth during this period is approximately double that of maxillary growth, and that males exhibit more prolonged and pronounced growth compared to females, who tend to reach skeletal maturity earlier. Furthermore, research tracking craniofacial changes from adolescence into adulthood confirms that skeletal and soft tissue adaptations are ongoing in the late teenage years, supporting the notion that individuals under 21 may not have achieved full craniofacial maturity [51].

A further result, interpretable in light of the limitations of our study, is that in both age groups, the total tooth volume was significantly greater than the superimposed volume, confirming a consistent volumetric discrepancy regardless of age. The magnitude of this difference was slightly higher in the <21 group, which may support the interpretation that incomplete skeletal maturation contributes to reduced morphological congruence between teeth and facial structures. Although the clinical relevance of this variation remains uncertain, these findings underscore that perfect morphological alignment between MCIs and facial morphology is rarely achieved, even in skeletally mature individuals.

Evidence-based sexual dimorphism in dental volume [54,55,56] is also highlighted by our gender-related analysis, which showed that men exhibited larger MCIs in absolute volumetric terms, while the degree of morphological alignment with the facial surface remained comparable across sexes. An additional observation was that, in women, a strong positive correlation emerged between total tooth volume and remaining volume, underlying that larger teeth tend to display greater volumetric divergence from the facial surface, which may reflect gender-specific craniofacial proportions or adaptive soft tissue dynamics within this Romanian young population segment. In contrast, this relationship was absent in men, suggesting that in males the congruence between tooth size and facial morphology is less influenced by volumetric variation. Furthermore, in both sexes, total tooth volume exceeded the superimposed volume, confirming a consistent volumetric discrepancy regardless of gender, with the magnitude of this difference slightly higher in women than in men. Although the clinical relevance of this variation should be approached with caution, these findings indicate that gender influences absolute tooth size and, to some extent, affects the degree of morphological congruence with facial structures.

The shape of the maxillary central incisor and its morphological correspondence with the face according to sex has been the subject of much debate to date, with most studies indicating that although men generally have larger maxillary central incisors and broader faces than women, there is no significant relationship between the shape of the maxillary central incisor and the shape of the face, regardless of sex. For instance, a Serbian study found no significant gender differences in the matching of incisor shape to dental arch or dental arch to face, though a minor difference was noted in the direct matching of tooth to face shape, with men showing slightly more impaired congruence [41]. Similarly, a cross-sectional study in Pakistan revealed that while men have longer incisors, facial profile does not affect incisor length, and gender does not influence the relationship between facial profile and incisor size [57]. Some population-specific studies have reported minor differences, but these are not consistent or strong enough to suggest a meaningful gender effect on incisor-face congruence [58,59,60], and moreover, to date, we have not identified studies that investigate or report that, in women, a larger volume of the maxillary central incisor leads to a decrease in congruence or correspondence with the face.

According to our study, the null hypothesis was confirmed; therefore, a 3D correspondence exists between the buccal surface morphology of the MCI and the corresponding face shape, whereas the alternative hypothesis was supported, meaning that complete morphological congruence does not occur. Beyond the interpretative dimension of these results, the present research introduces two major novel elements. First, the methodological approach based on high-resolution intraoral and facial scanning combined with volumetric superimposition represents a significant advancement over traditional 2D or proportion-based analyses, which have historically relied on linear measurements or photographic assessments. Unlike the conventional techniques, our protocol provides an objective, reproducible, and quantifiable metric of morphological correspondence, enabling clinicians and researchers to capture the 3D complexity of dentofacial relationships with unprecedented precision. Second, the findings reveal that larger MCIs tend to show greater remaining volume after superimposition, indicating reduced congruence with facial morphology. This strong correlation, observed particularly in women and individuals under 21 years, identifies volumetric size as a determinant of morphological incongruence—an aspect overlooked in previous studies focused only on shape or linear dimensions.

Despite these insights, there are also important limitations worth mentioning, such as the single-center investigation methodology, which restricts the generalizability of the findings to other populations with different ethnic, demographic, or clinical characteristics. Additionally, the sample consisted exclusively of first-year dental students, which introduces selection bias and limits the age distribution. Although young adulthood is generally defined as spanning approximately 18 to 35 years, our study included only participants aged 18–27, which narrows the variability within this life stage and also excludes older adults and adolescents.

Moreover, the analysis relied on static 3D scans, which do not capture dynamic facial expressions or soft tissue mobility that influence esthetic perception in real-life scenarios. Furthermore, although the alignment protocol was standardized and partially automated, it still depends on specific anatomical landmarks, which may introduce variability in cases with subtle asymmetries. Finally, the cross-sectional design prevents assessment of longitudinal changes in dentofacial relationships over time. Future research should address these limitations by including multi-center cohorts, more diverse populations, dynamic imaging techniques, and predictive models that integrate functional and esthetic parameters. Another limitation is the use of a homogeneous convenience sample from a single center (first-year dental students), which improves procedural consistency but restricts external validity. Craniofacial morphology and dentofacial relationships may differ across broader age strata and populations; therefore, generalization beyond young adults in this academic setting should be made cautiously. Future studies should validate these results in multi-center cohorts with wider demographic variability.

Volumetric analysis methods exhibit substantial heterogeneity in protocols for region-of-interest selection and volume calculation, preventing direct comparison across studies and precluding the establishment of reliable reference data. Common limitations include data dependency of AI algorithms, limited generalizability from single-center training datasets, and moderate-to-high risk of bias in existing studies [60,61,62,63]. The evidence supports clinical implementation of AI-assisted segmentation and landmarking for improved workflow efficiency, while facial prediction and volumetric analysis applications require further standardization and validation before widespread adoption.

Recent research [64,65,66,67,68,69,70] demonstrates that AI-based 3D technologies, particularly deep learning segmentation and automatic landmarking achieve high accuracy (92–97%) with dramatic time efficiency gains for clinical workflows, while 3D facial–dental integration accuracy depends critically on imaging modality and registration method, and volumetric analysis applications remain limited by substantial methodological heterogeneity that prevents cross-study comparison and standardized clinical implementation.

## 5. Conclusions

The comprehensive 3D analysis revealed that while a substantial correspondence exists between MCI morphology and facial structure, perfect alignment is inherently unattainable in a natural population, as evidenced by consistent volumetric discrepancies. Gender-related differences in dentofacial congruence indicate that, in men, variability in MCI volume exerts minimal influence on 3D facial correspondence, reflecting a relatively stable dentofacial relationship, whereas in women, increasing tooth volume is associated with progressively greater residual discrepancies after superimposition. A similar pattern in individuals younger than 21 years demonstrates reduced predictability of dentofacial correspondence, likely reflecting ongoing craniofacial maturation; consequently, in this age group, prosthetic restorative strategies should not rely exclusively on facial determinants but rather incorporate additional individualized reference criteria.

The integration of automated Blender/Python computational workflows with high-resolution 3D imaging enabled a reproducible and physiologically representative assessment of dentofacial relationships, effectively overcoming the inherent limitations of traditional 2D or linear methodologies. The volumetric size of the MCI acts as a determinant of morphological congruence with facial structures, reinforcing its predictive value in treatment planning. Facial scanning and 3D analysis of facial morphology thus provide a reliable starting point for individualized dental restorations of central incisors, particularly when other dental records are missing. Overall, these findings establish a robust and reproducible methodological framework for integrating dentofacial morphology in both research and clinical practice, underscoring the necessity of volumetric 3D evaluation and providing a contemporary basis for refining esthetic planning strategies and re-examining theoretical constructs such as the “law of harmony” within personalized dentistry.

## Figures and Tables

**Table 1:** Description of analyzed parameters.

Parameter	Nr.		Percent	
Gender (Female/Male)	64/34		65.3%/32.7%	
Age ≥ 21 years	46		47.9%	
Parameter	Mean ± SD	Median (IQR)	Min	Max
Age	20.56 ± 1.29	20 (20–21)	18	27
Total tooth volume (×1000)	1626.1 ± 2106.6	1634.3 (1452.3–1782.3)	1044.3	2070.3
Remaining tooth volume (×1000)	285.05 ± 137.01	240.6 (193.1–336.2)	95.53	961.7
Superimposed tooth volume (×1000)	1341 ± 1952.3	1355.4 (1192.2–1471.8)	811	1717.1
% Superimposition	82.64 ± 7.36	84.7 (79.73–87.88)	51.69	93.7

**Table 2:** Correlation between total tooth volume and remaining tooth volume.

Correlation	*p* *
Total tooth volume (*p* = 0.694 **) × Remaining tooth volume (*p* < 0.001 **)	<0.001, R = 0.448

* Spearman’s rho correlation coefficient, ** Shapiro–Wilk test.

**Table 3:** Comparison between the total tooth volume and superimposed tooth volume.

Tooth Volume (*1000)	Mean ± SD	Median (IQR)	*p* *
Total (*p* = 0.694 **)	1626.1 ± 2106.6	1634.3 (1452.3–1782.3)	<0.001
Superimposed (*p* = 0.292 **)	1341 ± 1952.3	1355.4 (1192.2–1471.8)	
Difference	Mean (95% C.I.) = 285,052.34 (257,583–312,521)		
Difference as %	Mean (95% C.I.) = 17.53% (15.84–19.22%)		

* Paired-sample *t*-test, ** Shapiro–Wilk test.

**Table 4:** Correlations between total tooth volume, superimposition %, age, and age group.

Correlation		*p* *	
Age x Total tooth volume		0.113, R = −0.163	
Age x Superimposition ***%		0.337, R = 0.099	
Age/Total volume	Mean ± SD	Median (IQR)	*p*
<21 years	1,652,082 ± 216,940	1,636,941 (1,522,912–1,806,715)	0.160 **
≥21 years	1,591,132 ± 203,241	1,589,833 (1,431,591–1,734,787)	
Age/Superimposition%	Mean ± SD	Median (IQR)	*p*
<21 years	82.54 ± 6.85	84.46 (80.04–86.75)	0.500 ***
≥21 years	82.68 ± 8.08	85.32 (76.73–88.15)	

* Spearman’s rho correlation, ** coefficient Student’s *t*-test, *** Mann–Whitney U Test.

**Table 5:** Comparison of the total tooth volume, superimposition % according to gender.

Gender/Total Volume	Mean ± SD	Median (IQR)	*p*
Female	1,583,561 ± 225,978	1,579,566 (1,405,585–1,737,006)	0.005 *
Male	1,706,232 ± 151,086	1,667,763 (1,586,926–1,827,369)	
Gender/Superimposition%	Mean ± SD	Median (IQR)	*p*
Female	82.27 ± 7.69	84.34 (79.55–87.69)	0.526 **
Male	83.33 ± 6.73	85.01 (80.08–88.35)	

* Student’s *t*-test, ** Mann–Whitney U test.

**Table 6:** Correlation between the total tooth volume and the remaining tooth volume according to gender.

Correlation		*p* *
Total tooth volume (*p* = 0.428 **) x Remaining tooth volume (*p* < 0.001 **)	Women	<0.001, R = 0.515
Total tooth volume (*p* = 0.686 *) x Remaining tooth volume (*p* = 0.041 **)	Men	0.310, R = 0.179

* Spearman’s rho correlation coefficient, ** Shapiro–Wilk test.

**Table 7:** Comparison between the total tooth volume and superimposed tooth volume according to gender.

Tooth Volume (*1000)		Mean ± SD	Median (IQR)	*p* *
Total (*p* = 0.428 **)	Women	1583.3 ± 225.9	1579.5 (1405.5–1737)	<0.001
Superimposed (*p* = 0.865 **)		1298.2 ± 194.8	1288.1 (1178.2–1443.3)	
Difference		Mean (95% C.I.) = 285,296.23 (248,004–322,587)		
Difference as %		Mean (95% C.I.) = 18.02% (15.66–20.37%)		
Tooth volume (*1000)		Mean ± SD	Median (IQR)	*p* *
Total (*p* = 0.686 **)	Men	1706.2 ± 151	1667.7 (1586.9–1827.3)	<0.001
Superimposed (*p* = 0.102 **)		1421.6 ± 171.2	1452.8 (1330.5–1554)	
Difference		Mean (95% C.I.) = 284,593.25 (245,379–323,807)		
Difference as %		Mean (95% C.I.) = 16.68% (14.38–18.98%)		

* Paired-sample *t*-test, ** Shapiro–Wilk test.

**Table 8:** Correlation between the total tooth volume and remaining tooth volume in participants and age groups.

Correlation		*p* *
Total tooth volume (*p* = 0.419 **) × Remaining tooth volume (*p* < 0.001 **)	age < 21 years	<0.001, R = 0.662
Total tooth volume (*p* = 0.711 *) × Remaining tooth volume (*p* < 0.001 **)	≥21 years	0.251, R = 0.173

* Spearman’s rho correlation coefficient, ** Shapiro–Wilk test.

**Table 9:** Comparison between the total tooth volume, superimposed tooth volume, and age group.

Tooth Volume (*1000)		Mean ± SD	Median (IQR)	*p* *
Total (*p* = 0.711 **)	age < 21 years	1591.1 ± 203.2	1589.8 (1431.5–1734.7)	<0.001
Superimposed (*p* = 0.518 **)		1316.1 ± 213	1346.9 (1166.7–1481.7)	
Difference		Mean (95% C.I.) = 275,001.23 (234,909–315,092)		
Difference as %		Mean (95% C.I.) = 17.28% (14.76–19.80%)		
Tooth volume (*1000)		Mean ± SD	Median (IQR)	*p* *
Total (*p* = 0.419 **)	age ≥ 21 years	1652 ± 216.9	1636.9 (1522.9–1806.7)	<0.001
Superimposed (*p* = 0.628 **)		1357.9 ± 178.6	1355.4 (1249.1–1471)	
Difference		Mean (95% C.I.) = 294,131.51 (253,799–334,463)		
Difference as %		Mean (95% C.I.) = 17.80% (15.36–20.24%)		

* Paired-sample *t*-test, ** Shapiro–Wilk test.

## Data Availability

The original contributions presented in the study are included in the article; further inquiries can be directed to the corresponding authors.

## Abbreviations

The following abbreviations are used in this manuscript:

AI	Artificial Intelligence
CAD	Computer-Aided Design
CAD/CAM	Computer-Aided Design/Computer-Aided Manufacturing
DSD	Digital Smile Design
IQR	Interquartile Range
MCI	Maxillary Central Incisor
RED	Recurrent Esthetic Dental
RTV	Remaining Tooth Volume
STL	Stereolithography
V_total	Total Volume of the Extruded Buccal Surface of the MCI
V_superimposed	Superimposed Volume of the Tooth Segment Intersecting with the Facial Mesh
%SI	Percentage of Superimposition
